# Post-translational modifications are key players of the *Legionella pneumophila* infection strategy

**DOI:** 10.3389/fmicb.2015.00087

**Published:** 2015-02-10

**Authors:** Céline Michard, Patricia Doublet

**Affiliations:** ^1^Legionella Pathogenesis Group, International Center for Infectiology Research, Université de LyonLyon, France; ^2^INSERM U1111Lyon, France; ^3^Ecole Normale Supérieure de LyonLyon, France; ^4^Centre International de Recherche en Infectiologie, Université Lyon 1Lyon, France; ^5^Centre National de la Recherche Scientifique, UMR5308Lyon, France

**Keywords:** post-translational modification, *Legionella pneumophila*, Dot/Icm effectors, host cell pathways hijacking, *Legionella* virulence

## Abstract

Post-translational modifications (PTMs) are widely used by eukaryotes to control the enzymatic activity, localization or stability of their proteins. Traditionally, it was believed that the broad biochemical diversity of the PTMs is restricted to eukaryotic cells, which exploit it in extensive networks to fine-tune various and complex cellular functions. During the last decade, the advanced detection methods of PTMs and functional studies of the host–pathogen relationships highlight that bacteria have also developed a large arsenal of PTMs, particularly to subvert host cell pathways to their benefit. *Legionella pneumophila*, the etiological agent of the severe pneumonia legionellosis, is the paradigm of highly adapted intravacuolar pathogens that have set up sophisticated biochemical strategies. Among them, *L. pneumophila* has evolved eukaryotic-like and rare/novel PTMs to hijack host cell processes. Here, we review recent progress about the diversity of PTMs catalyzed by *Legionella*: ubiquitination, prenylation, phosphorylation, glycosylation, methylation, AMPylation, and de-AMPylation, phosphocholination, and de-phosphocholination. We focus on the host cell pathways targeted by the bacteria catalyzed PTMs and we stress the importance of the PTMs in the *Legionella* infection strategy. Finally, we highlight that the discovery of these PTMs undoubtedly made significant breakthroughs on the molecular basis of *Legionella* pathogenesis but also lead the way in improving our knowledge of the eukaryotic PTMs and complex cellular processes that are associated to.

## INTRODUCTION

Post-translational modifications (PTMs) are widely used by eukaryotes to control quickly, locally and specifically the enzymatic activity, localization or stability of their proteins, and thus to fine-tune key factors of the cellular biology to environmental changes. Eukaryotic PTMs involve diverse modifications of specific residues of the protein by the covalent addition of simple or complex chemical groups; they include the addition of chemical group (e.g., phosphate, methyl, or acetate), more complex molecules (e.g., carbohydrates or lipids), the covalent linkage of small proteins (e.g., ubiquitin), and the irreversible hydrolysis of a specific peptide bond between two amino acids, or proteolysis (for review, see Walsh et al., 2005). PTMs are catalyzed by specific enzymes and most of them are reversed by antagonistic catalytic activities. Traditionally, it was believed that the broad biochemical diversity of the PTMs is restricted to complex eukaryotic cells, which exploit it in extensive networks to control various and complex cellular functions. During the last decade, the advanced detection methods of PTMs, including the modified peptides enrichment combined with high accuracy mass spectrometry, the pathogen genomes sequencing that predicts PTMs activities, and the functional studies of the host–pathogen relationships highlight that bacteria have also developed a large arsenal of PTMs, particularly to subvert host cell pathways to their benefit, to escape to the host defences, and finally to promote their replication (for review, see Ribet and Cossart, 2010a,b).

*Legionella pneumophila*, the etiological agent of the severe pneumonia legionellosis, is a paradigm of highly adapted intravacuolar pathogens that have set up sophisticated biochemical strategies to hijack host cell processes. *Legionella* pathogenic strains (i) emerge from the environment after intracellular multiplication in protozoans, especially in amoebae; (ii) are disseminated by contaminated aerosols; and (iii) can infect alveolar macrophages of its accidental human host. Within environmental phagocytic cells and human macrophages, *L. pneumophila* evades endocytic degradation (Horwitz and Maxfield, 1984; Clemens et al., 2000), controls the innate immune response, especially the NF-κB pathway (Schmeck et al., 2007; Shin et al., 2008), and triggers the biogenesis of a *Legionella*-containing vacuole (LCV), a rough endoplasmic reticulum-like compartment permissive for its intracellular replication (Horwitz, 1983; Kagan and Roy, 2002). Crucial for hijacking host cell vesicle trafficking necessary for LCV biogenesis, and subsequently for intracellular multiplication of *L. pneumophila,* is the Dot/Icm Type 4 Secretion System (T4SS; Marra et al., 1992; Andrews et al., 1998) that translocates into the host cell cytosol over 275 bacterial proteins, named effectors (Zhu et al., 2011). Many Dot/Icm effectors harbor eukaryotic domains (Cazalet et al., 2004), such as protein–protein interaction domains and enzymatic activity-associated domains, in particular for PTMs such as methylation, phosphorylation, ubiquitination, and glycosylation, which support that *L. pneumophila* has evolved eukaryotic-like PTMs to hijack host cell processes.

Here, we review recent progress about the diversity of PTMs catalyzed by *Legionella*. We focus on the host cell pathways targeted by the bacteria-catalyzed PTMs and we stress the importance of the PTMs in the *Legionella* infection strategy.

## DIVERSITY OF PTMs CATALYZED BY *L. pneumophila*

A key finding of the *L. pneumophila* genome analysis was the identification of a large number of proteins similar to eukaryotic proteins. The wide variety of these proteins includes enzymatic activity–associated domains for various PTMs such as phosphorylation, glycosylation, methylation, prenylation, ubiquitination, reversible AMPylation, and phosphocholination of host cell proteins to modulate cellular functions (**Table 1**).

**Table 1:** Diversity of PTMs catalyzed by Dot/Icm effectors of *Legionella pneumophila.*

PTMs	Mechanism	Effector name	Motif	Target	Reference
Phosphorylation	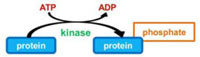
		LegK1	STPK	IκB	Ge et al. (2009); Hervet et al. (2011)
		LegK2	STPK		Hervet et al. (2011)
		LegK3	STPK		Hervet et al. (2011)
		LegK4	STPK		Hervet et al. (2011)
Methylation	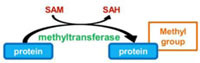	LegAS4/RomA	SET domain	H3	Li et al. (2013), Rolando et al. (2013b)
Prenylation	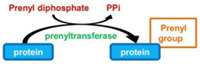	AnkB/LegAU13/Ceg27	CAAX		Price et al. (2010a)
Ubiquitination	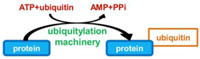
		LubX/LegU2	U-box	Clk1 /SidH	Kubori et al. (2008)
		AnkB/LegAU13 /Ceg27	F-box	Skp1/ParvB	Price et al. (2009), Lomma et al. (2010)
		LegU1	F-box	BAT3	Ensminger and Isberg (2010)
		SidC			Hsu et al. (2014)
Glycosylation	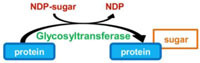
		Lgt1	Coiled- coil	eEF1A	Belyi et al. (2006)
		Lgt2	Coiled- coil	eEF1A	Belyi et al. (2008), Aktories (2011)
		Lgt3/Legc5	Coiled- coil	eEF1A	Belyi et al. (2008), Aktories (2011)
		SetA			Heidtman et al. (2009)
AMPylation	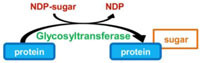	SidM/DrrA		Rab1	Müller et al. (2010)
DeAMPylation	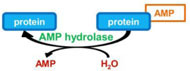	SidD		Rab1	Neunuebel et al. (2011); Tan et al. (2011)
Phosphocholination	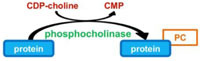	AnkX /AnkN /LegA8	Ankyrin	Rab1	Mukherjee et al. (2011)
Dephosphocholination	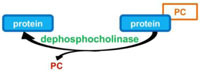	Lem3		Rab1	Tan et al. (2011)

*STPK, Ser/Thr protein kinase*.

### PROTEIN PHOSPHORYLATION

Phosphorylation–dephosphorylation of proteins represents a powerful regulatory mechanism of cellular activity. Indeed, intensive research has revealed that eukaryotes contain numerous interconnected signal transduction networks in which protein phosphorylation plays a dominant role for controlling essential functions, such as growth, cell cycle and apoptosis, in response to extracellular stimuli and stresses. It consists in the reversible covalent addition of a phosphate group, from the phosphate donor ATP, to specific residues of a target protein, the most frequent being hydroxyl groups of serine, threonine or tyrosine residues. The phosphoester bond is catalyzed by eukaryotic protein kinases that share a common catalytic domain characterized by 11 conserved Hanks’s subdomains (Hanks, 2003). Conversely, phosphatases hydrolyze the phosphoester bond, thereby releasing the phosphate group and restoring the acceptor amino acid in its unphosphorylated form.

The genomes of the six sequenced *L. pneumophila* strains, Philadelphia, Lens, Paris, Corby, Alcoy, and 130b, have been reported to encode four putative eukaryotic-like serine/threonine kinases, named LegK1–LegK4 (Cazalet et al., 2004; de Felipe et al., 2005; D’Auria et al., 2010; Schroeder et al., 2010). Alignment with several eukaryotic protein kinases revealed residues that are highly conserved in the Hanks’ subdomains, including the glycine-rich loop and the invariant lysine in subdomains I and II, which are essential for binding and correct orientation of the phosphate donor ATP. *In vitro* phosphorylation assays confirmed that these kinases were functional for autophosphorylation and/or phosphorylation of the classical substrate for eukaryotic kinases Myelin-basic protein (Hervet et al., 2011; **Table 1**).

### PROTEIN ALKYLATION

Protein alkylation consists in the addition of alkyl substituents on specific amino acids. The common alkyl groups transferred are the methyl (C1) or the C15 (farnesyl)/C20 (geranyl–geranyl) isoprenyl groups, leading to protein methylation and protein prenylation, respectively.

Protein methylation typically takes place on arginine or lysine residues in the protein sequence. Arginine can be methylated once or twice, with either both methyl groups on one terminal nitrogen (asymmetric dimethylated arginine) or one on both nitrogens (symmetric dimethylated arginine) by peptidylarginine methyltransferases (PRMTs). Lysine can be methylated once, twice, or three times by lysine methyltransferases (Walsh et al., 2005). Protein methylation has been extensively studied in the histones. The transfer of methyl groups from S-adenosyl methionine (SAM) to histones is catalyzed by SET domain-containing proteins. This protein family is characterized by an ∼130 amino acid-long SET domain that possesses catalytic activity toward the ε-amino group of lysine residues. *In vivo*, lysine methylation can be dynamically regulated by the opposing actions of lysine methyltransferases and lysine demethylases (Herz et al., 2013). *L. pneumophila* genome analysis revealed that all the five strains Philadelphia, Lens, Paris, Corby, and Alcoy encode each an orthologous protein encoding a SET domain that show 95–100% sequence identity over the entire length (Cazalet et al., 2004). *In vitro* assays recently demonstrated that Lpp1683 in Paris strain and Lpg1718 in Philadelphia strain, display histone methyltransferase activity toward the histone H3 substrate (Li et al., 2013; Rolando et al., 2013b; **Table 1**).

Prenylation, i.e., addition of a farnesyl (C15) or a geranyl–geranyl (C20) group, is a PTM that covalently links a lipid moiety at the cysteine residue of the CAAX motif in the C-terminal region of proteins (where C represents cysteine and A an aliphatic amino acids). The Ras GTPases, Rab small GTPases, and protein kinases superfamilies have members that can be prenylated on cysteine thiolate side chains. The lipid anchors drive the modified proteins to partition more to membranes, thus controlling their subcellular localization (Walsh et al., 2005). Interestingly, bioinformatic approaches identified 11–12 (depending on the strains) different *Legionella* proteins containing a CAAX motif in the C terminus, which have been so called Pel proteins for Prenylated effectors of *Legionella* (Ivanov et al., 2010; Price et al., 2010a,b). Six of these proteins had highly conserved homologs across all *Legionella* stains, whereas four of the proteins were unique for either the Philadelphia or Lens strain. Host farnesyltransferase and class I geranylgeranyltransferase were both involved in the lipidation of the *Legionella* CAAX motif proteins, among which AnkB from *L. pneumophila* AA100 and Philadelphia Lp01 (Ivanov et al., 2010; Price et al., 2010a,b; **Table 1**).

### PROTEIN UBIQUITINATION

**FIGURE 1:**
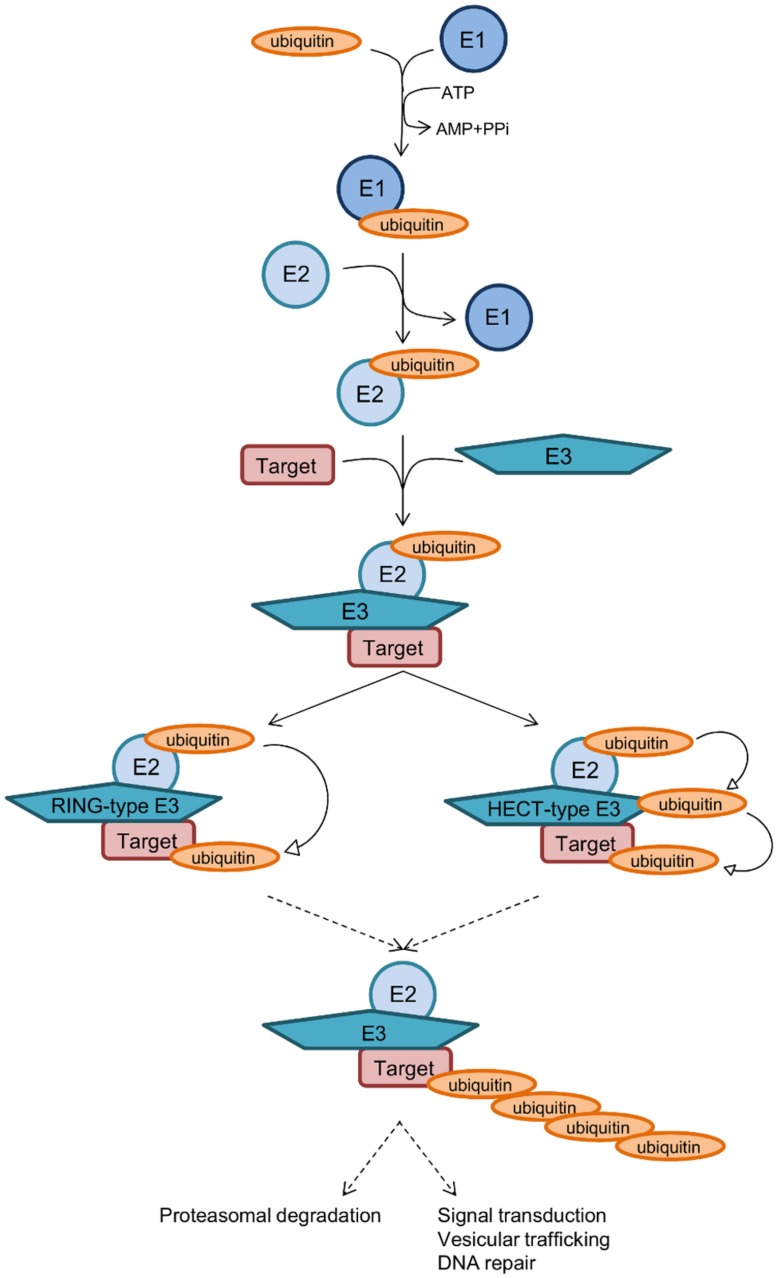
**Sequential steps of ubiquitination.** The small protein ubiquitin is first activated by an E1 enzyme in an ATP-dependent manner, then transferred to an E2 conjugating enzyme. Two main classes of E3 enzymes, the RING, and HECT classes, transfer differently the ubiquitin to a specific protein substrate. Polyubiquitin chains are then built up through Lys48 side chains or through Lys63 of ubiquitin, which directs the protein to proteasome degradation or participate in signal transduction, vesicular trafficking, or DNA repair.

Ubiquitination consists in the addition of one or several ubiquitins on a target protein, most frequently on lysine residue, although linkages on cysteine, serine or threonine, or on the N-terminal amino group have also been reported. Ubiquitin is a small protein of 9 kDa, which contains itself seven lysines; all of these lysine residues can be used as a target for the addition of another ubiquitin moiety, thus leading to polyubiquitination. Polyubiquitin chains built up through Lys48 side chains are commonly associated with proteasome binding and degradation of the modified protein, whereas chains tethered through Lys63 participate in signal transduction, vesicular trafficking or DNA repair (Hochstrasser, 2009). The conjugation of ubiquitin requires different enzymes (**Figure 1**): E1 activating enzymes that bind ubiquitin in a ATP dependent manner; E2 conjugation enzymes that bind ubiquitin in a thioester bond; E3 ubiquitin ligases are then required to catalyze the efficient transfer of the activated ubiquityl protein tag to Lys side chains of target proteins. There are two different families of E3 ubiquitin ligases, the HECT family and the RING/U-Box family. In the RING family, some of the enzyme 3 are multicomponent catalysts, such as the SCF E3s that consist in four subunits: the invariable subunit Skp1, the central core component Cullin, the RING finger protein Rbx1/Roc1 and the variable F-Box protein that serves as a receptor for the target protein, such providing selectivity for a given protein (Lorick et al., 1999). There are several 100 isoforms of such E3 ubiquitin ligases in higher eukaryotes, which allow subtle discrimination among many target proteins selected for ubiquitination.

Genome analysis of the *L. pneumophila* strains Paris and Philadelphia revealed they encode a protein, named LubX, containing two U-box domains similar to eukaryotic E3 ubiquitin ligases (Cazalet et al., 2004). Indeed, *in vitro* and in host cells, LubX functions as an ubiquitin ligase (Kubori et al., 2008). Moreover, *L. pneumophila* encodes several proteins with predicted F-Box motifs (de Felipe et al., 2005). The best characterized is the AnkB protein, that is conserved in the five sequenced *L. pneumophila* strains (**Table 1**). Genome sequence of *L. pneumophila* Philadelphia reveals the presence of at least another four F-box containing proteins (Price and Kwaik, 2010). These include Lpg2224 (PpgA), Lpg2525 (MavK), LicA, and LegU1. Finally, the Dot/Icm effector SidC has been recently reported to define a unique family of ubiquitin ligase (Hsu et al., 2014). While the amino acid sequence of SidC does not exhibit significant homology with any known protein, the crystal structure of its N-terminal domain revealed a canonical catalytic triad C46-H444-D446 found in cystein-based proteases and deubiquitinases. Unexpectedly, *in vitro* assays demonstrated that SidC exhibits ubiquitin ligase rather than protease or deubiquitinase activity. More precisely, SidC catalyzes the formation of high–molecular-weight ubiquitinated conjugates in a manner that is dependent on the catalytic residue C46. Authors further showed that the SidC paralog SdcA has also ubiquitin ligase activity (Hsu et al., 2014; **Table 1**).

### PROTEIN GLYCOSYLATION

*O*-glycosylation and *N*-glycosylation of proteins are very common in eukaryotes but only *O*-glycosylation has been described to date in the two major groups of bacterial toxins. *O*-glycosylation consists in the modification of serine or threonine residues. A 60 kDa protein that exihibited UDP-glycosyltransferase activity toward a 50 kDa protein from HeLa cell lysates was firstly purified from *L. pneumophila* (Belyi et al., 2003). This protein, named Lgt1 (for *Legionella* glycosyltransferase 1) contains a DXD motif, which is conserved in many prokaryotic and eukaryotic glucosyltransferases. In *L. pneumophila* strains Philadelphia, Lens, Paris, and Corby, two other proteins very similar to Lgt1 were then identified; they were thus called Lgt2 and Lgt3 and were shown to exhibit the same glycosylase activity (Belyi et al., 2008; Aktories, 2011). An additional protein, namely SetA, possesses a functional glycosyltransferase domain (Heidtman et al., 2009). However, its target in the host cell has not been yet identified (**Table 1**).

### REVERSIBLE PROTEIN AMPylation

AMPylation or adenylylation is the addition of an adenosine monophosphate (AMP) group from ATP onto a threonine, tyrosine, or serine residue of a protein. This PTM was first and recently discovered on host cell proteins infected by *Vibrio parahaemolyticus* and *Histophilus somni* (Worby et al., 2009; Yarbrough et al., 2009). This activity involves a conserved domain, called Fic domain (for filamentation induced by cAMP domain), which was originally described in *Escherichia coli* as a stress response protein associated with filamentous bacterial growth in the presence of cAMP (Komano et al., 1991). The Fic domain is also found in eukaryotic proteins, and AMPylation has now been shown to be naturally occurring in eukaryotic cells (Kinch et al., 2009; Roy and Mukherjee, 2009; Worby et al., 2009; Yarbrough and Orth, 2009; Yarbrough et al., 2009). Despite the lack of a consensus Fic domain on its sequence and that its amino acid sequence did not suggest its function, the protein SidM from *L. pneumophila* has been recently shown to possess AMPylase activity (Müller et al., 2010). More precisely, its N-terminal domain exhibits structural similarities with the C-terminal domain of the glutamine synthase adenylyl transferase, which leads the authors to speculate that the N-terminal region of SidM might have AMPylase activity toward the small GTPase Rab1, the substrate of its GEF domain. Indeed, *in vitro* assays and mass spectrometry analysis demonstrates that SidM, more precisely its N-terminal domain, AMPylates Rab1 on the Tyr77 residue (Müller et al., 2010; **Table 1**).

AMPylation is a reversible process. Two independent groups simultaneously identified SidD from *L. pneumophila* as the first protein exhibiting a deAMPylase activity, by using two distinct approaches. Neunuebel et al. (2011) observed that a whole cell lysate from *L. pneumophila* but not from *E. coli* efficiently removes *in vitro* radiolabeled AMP from AMPylated Rab1. Given that genes of functionally linked proteins tend to be clustered on bacterial genomes, they deleted the immediately nearby *sidM* gene, namely *sidD* gene, and they observed that the whole cell lysate from the *sidD* mutant was not able anymore to deAMPylate SidM (Neunuebel et al., 2011). On the other hand, Tan and Luo identified SidD as a protein capable of suppressing the toxicity of the AMPylase SidM to yeast (Tan and Luo, 2011). Both groups demonstrated that SidD removes the AMP moiety from Tyr77 of Rab1, thus reversing the effect of SidM on this small GTPase activity (**Table 1**).

### REVERSIBLE PHOSPHOCHOLINATION

As mentioned above, the Fic domain is associated to enzymes that trigger AMPylation of target proteins. *In silico* analysis revealed that another protein from *L. pneumophila,* namely AnkX, contains a Fic domain. However, mass spectrometry demonstrated that AnkX promotes a novel PTM, namely phosphocholination rather than AMPylation (Mukherjee et al., 2011). Phosphocholination consists in the covalent link of a phosphocholine group to a serine residue (**Table 1**). More precisely, AnkX catalyzes the phosphocholination of Ser76 of the small GTPase Rab1, immediately upstream the Tyr77 AMPylated by SidM (Mukherjee et al., 2011).

Like AMPylation, phosphocholination is a reversible PTM. Tan et al. (2011) recently identified the Dot/Icm effector Lem3 as a protein capable to rescue the growth of yeast transformed by AnkX expression vector, which suggested that Lem3 was able to antagonize the activity of phosphocholination of AnkX. Indeed, *in vitro* assays demonstrated that Lem3 reverses the AnkX-dependent phosphocholination of Rab1 by removing the phosphocholine moiety from Rab1 (Tan et al., 2011; **Table 1**).

## PTMs FOR *Legionella* CONTAINING VACUOLE BIOGENESIS

**FIGURE 2:**
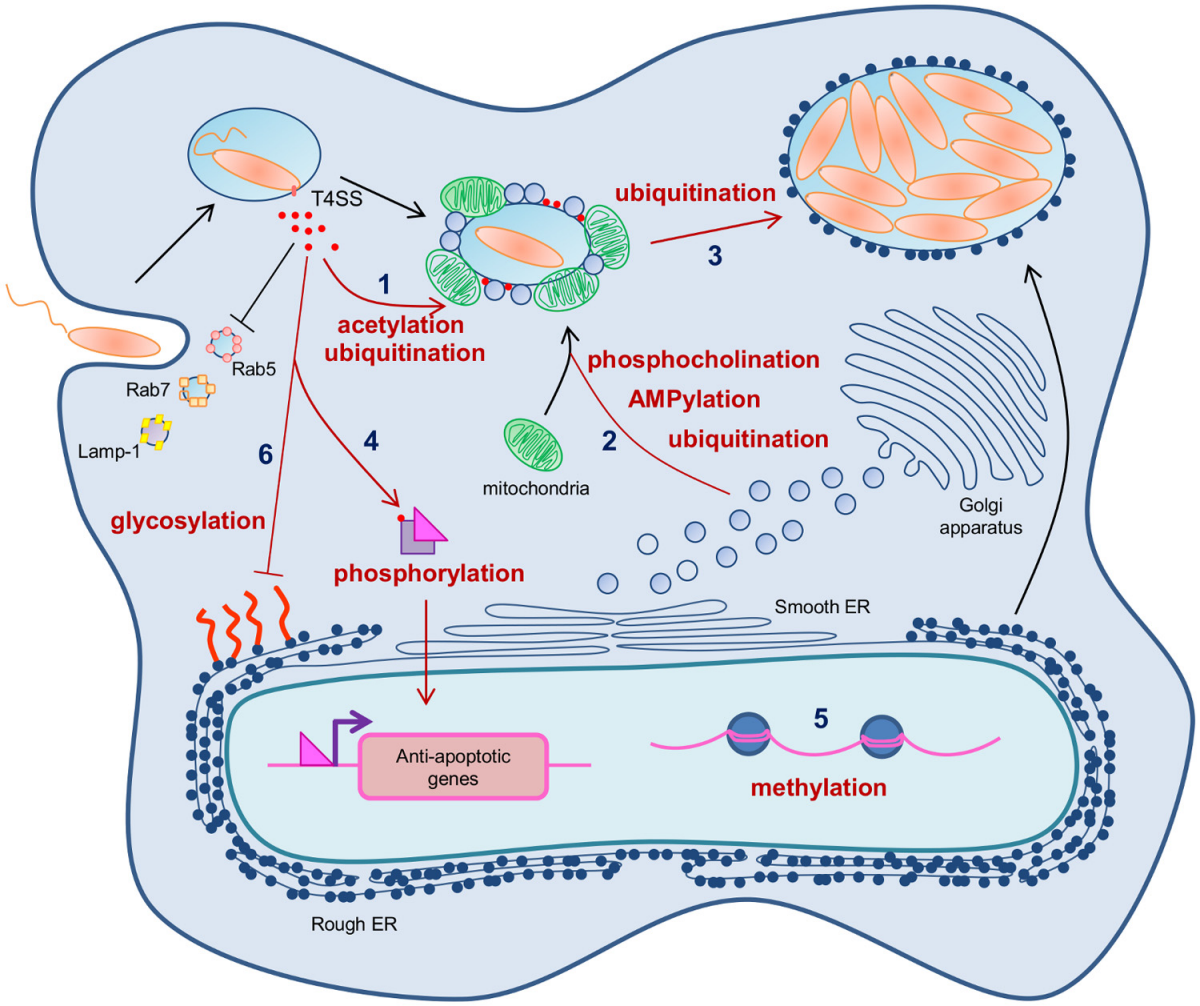
**Post-translational modifications controlling various infectious cycle steps of *Legionella pneumophila*.** Immediatly after uptake of the bacteria, *L. pneumophila* secretes a high number of Dot/Icm effectors into the host cell cytosol. (1) *L. pneumophila* exploits the host prenylation apparatus to alkylate some of these effectors and target them to the LCV surface. The effector LubX is able to ubiquitinate some effectors thus addressing them to proteasomal degradation and controlling their temporal presence on the LCV during infection. (2) Four Dot/Icm effectors reversibly AMPylate and phosphocholinate the host Rab1 small GTPase, thus controlling its activity to promote ER recruitment on the LCV, a prerequisite feature to make the phagosome a replicative niche. SidC and its paralog SdcA monoubiquitinate Rab1 and catalyze polyubiquitin chains formation necessary for ER recruitment on the LCV. (3) The Dot/Icm effector AnkB functions as a platform for the docking of polyubiquitinated proteins to the LCV membrane, thus promoting proteasome-mediated generation of free amino acids essential as energy and carbon sources for *L. pneumophila* intracellular proliferation. (4) The Dot/Icm effector LegK1 phosphorylates IκB thus mimicking the host IKKs, and triggering the activation of the NF-κB pathway and the transcription of NF-κB dependent genes. (5) The Dot/Icm effector LegAS4/RomA trigger the methylation of the histone H3, thus inducing epigenetic changes and subsequent transcriptional control of host genes. (6) Several Dot/Icm effectors exhibits glycosidase activity toward eEF1A, thus inhibiting the host cell translation.

*Legionella*-containing vacuole biogenesis is a main trait of *Legionella* intracellular fate that allows the bacteria to generate a niche permissive for intracellular replication. Within 15 min of uptake, the LCV is surrounded and fused with ER-derived smooth vesicles and mitochondria (Horwitz, 1983), and 4 h post-contact it is decorated by host cell ribosomes (Horwitz and Silverstein, 1981; Roy and Tilney, 2002), thus resulting in a replication-permissive vacuole (**Figure 2**). *Legionella*-containing vacuole biogenesis mobilizes complex molecular mechanisms that are strictly dependent on the Dot/Icm T4SS and its exceptionally high number of effectors. PTMs of both host cell proteins and Dot/Icm effectors play a key role in the fine-tuned orchestration of this infection step.

### Dot/Icm EFFECTORS ACETYLATION AND UBIQUITINATION SPATIO-TEMPORALLY CONTROL THEIR RECRUITMENT ON THE LCV

Given the high number of effectors, it could be assumed that both translocation into the host cell cytosol, organelles addressing, and degradation of each effector must be controlled such that it could sequentially participate to the LCV biogenesis. Many Dot/Icm effectors are targeted to the LCV surface. *L. pneumophila* uses [PI(4)P] to anchor some Dot/Icm substrates, such as SidC and SidM to the cytoplasmic face of LCV (Ragaz et al., 2008; Brombacher et al., 2009). Another way for *L. pneumophila* to address injected effectors to the LCV membrane, is the exploitation of the host cell prenylation apparatus (Ivanov et al., 2010; Price et al., 2010a). The Dot/Icm substrate AnkB, of strains *L. pneumophila* AA100 and Philadelphia Lp01, contains a CAAX motif. During infection, the CAAX motif of AnkB is modified by the host farnesylation machinery (Ivanov et al., 2010; Price et al., 2010a). Expression of a CAAX substituted-variant results in defective anchoring of AnkB to the LCV, severe defects in intracellular replication, and attenuation of intrapulmonary proliferation in a mouse model, thus demonstrating that the farnesyl-dependent vacuolar location of AnkB is essential to its role in the infectious cycle of *L. pneumophila* (Price et al., 2009).

In addition to the appropriate addressing of Dot/Icm effectors, a specific temporal control of their stability in the host cell is carried out during *L. pneumophila* infection. In that purpose, *L. pneumophila* interferes with the ubiquitin system to address some effectors to proteasomal degradation. LubX is a Dot/Icm effector containing two U-box domains and functions as a E3 ubiquitin ligase toward the cellular Clk1 protein. However, cellular consequences of ubiquitination of Clk1 remain unknown (Kubori et al., 2008). LubX was also shown to bind and polyubiquitinate *in vitro* SidH, another Dot/Icm effector. It mediates proteasomal degradation of SidH in infected cells. Thus, LubX is considered like a metaeffector that controls in space and time, the presence of another effector, by using ubiquitination PTM (Kubori et al., 2010).

### AMPylation AND PHOSPHOCHOLINATION CONTROLS THE GTPase Rab1 ACTIVATION FOR ER RECRUITMENT ON THE LCV

**FIGURE 3:**
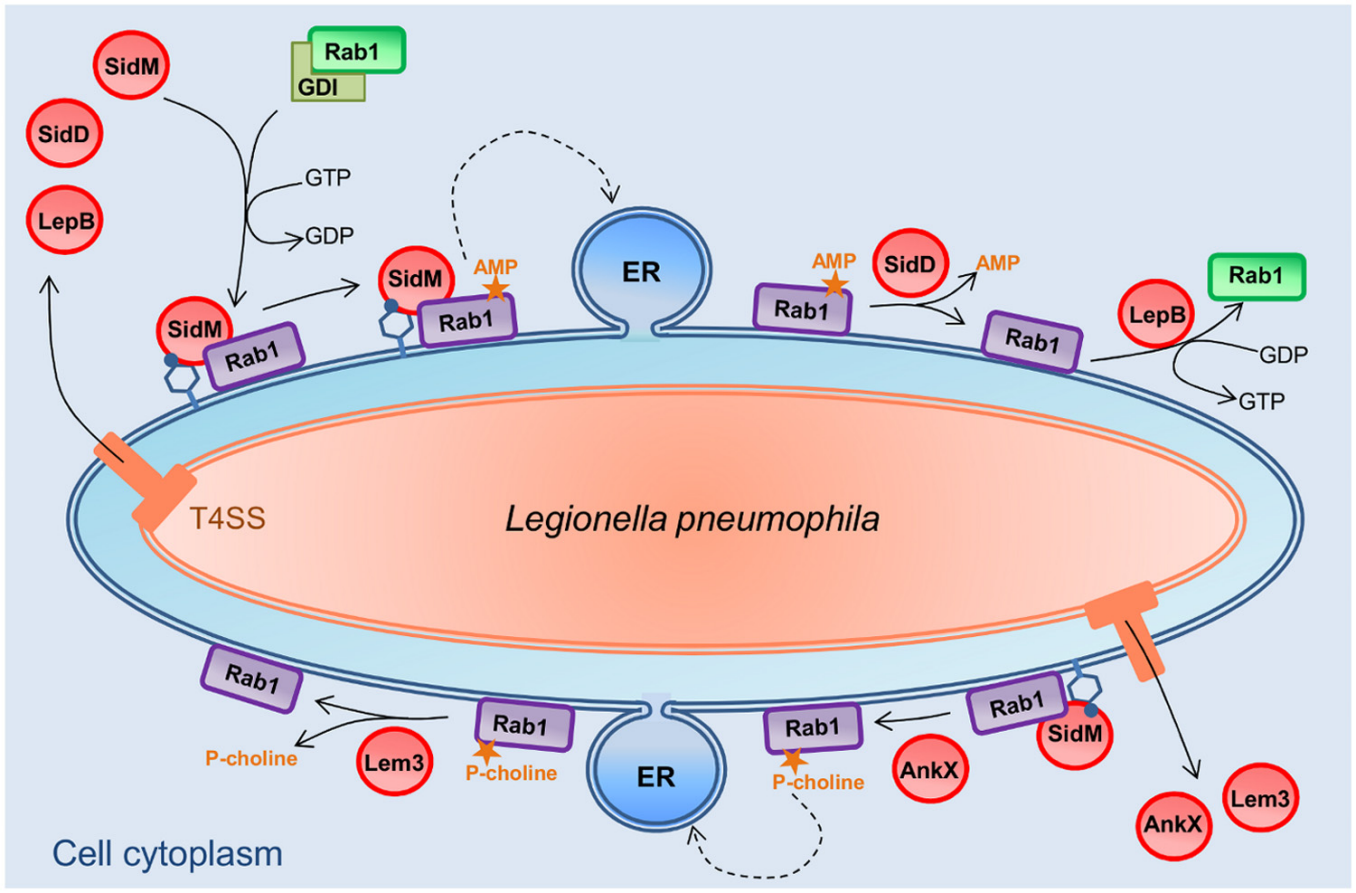
**Post-translational modifications of small GTPase Rab1 by *L. pneumophila* for LCV biogenesis.** The ER recruitment on the LCV is orchestrated by four Dot/Icm effectors-mediated PTMs. SidM releases Rab1 from GDI with its GEF activity. SidM then modifies Rab1 by AMPylation, i.e., the addition of a AMP moiety. This PTM locks Rab1 in the GTP-bound active state, and finally allows ER recruitment at the surface of the LCV. SidD removes AMP from Rab1, making it accessible for GAP activities, such as that exhibited by LepB. LepB promotes GTP hydrolysis of Rab1, removing it from the LCV. AnkX harbors a novel PTM activity, the phosphocholination, that transfers a phosphocholine moiety to Rab1, resulting in the same effect that the SidM-mediated AMPylation, i.e., locking Rab1 in the active form. The Dot/Icm effector Lem3 possesses an antagonistic activity to that of AnkX by removing the phosphocholine from Rab1.

One main characteristic of the LCV is that it is fused with ER-derived vesicles. The manipulation of host cell vesicular trafficking by *L. pneumophila* is strictly dependent of the Dot/Icm T4SS. In particular, some of Dot/Icm substrates target host cell small GTPases. Among them, the effector SidM interacts with Rab1, and its GEF and GDF activities result in Rab1 release from GDI (Ingmundson et al., 2007), and in LCV membrane associated GTP-coupled Rab1 (Arasaki et al., 2012), respectively (**Figure 3**). An additional PTM-associated enzymatic activity of the multifunctional protein SidM has recently been revealed. The N-terminal domain of SidM, which exhibits similarities with the catalytic domain of glutamin synthetase adenylyl transferase, modifies the tyrosine 77 of Rab1 by AMPylation or adenylylation, i.e., the addition of a AMP moiety (Müller et al., 2010). This PTM inhibits GAP-stimulated GTP hydrolysis, thus locking Rab1 in the GTP-bound active state, and finally allows ER recruitment at the surface of the LCV (**Figure 3**).

The activation of Rab1 by SidM is counteracted by two others Dot/Icm effectors, SidD and LepB (**Figure 3**). SidD removes AMP from Tyr77 of Rab1 (Neunuebel et al., 2011; Tan and Luo, 2011) by a protein phosphatase-like catalytic mechanism, as suggested by structural analysis (Rigden, 2011). DeAMPylation of Rab1 makes it accessible for GAP activities, such as that exhibited by LepB. Despite any similarity with eukaryotic Rab-GAPs, LepB harbors a Rab1-specific GAP activity that promotes GTP hydrolysis and subsequent removal of Rab1 from the LCV (Ingmundson et al., 2007; Mihai Gazdag et al., 2013). Consistent with the SidD-dependent action of LepB, the phenotype of a *lepB* mutant is similar to that of a *sidD* mutant, i.e., a prolonged localization of Rab1 on the LCV (Neunuebel et al., 2011).

Two additional Dot/Icm effectors target the Rab1 GTPase for PTM and participate to the temporal control of its activation during *Legionella* infection cycle. AnkX harbors a novel PTM activity, namely phosphocholination, that transfers a phosphocholine moiety from CDP-choline to serine 76 of Rab1, preceding the SidM-modified tyrosine (Mukherjee et al., 2011; **Figure 3**). Although the biological effect of this PTM of Rab1 is not completely deciphered, it results in the same biochemical consequence as the SidM-mediated AMPylation, i.e., locking Rab1 in the active form. Like AMPylation, phosphocholination is reversible. The Dot/Icm effector Lem3 has been recently shown to possess an antagonistic activity to that of AnkX by removing the phosphocholine from the Ser76 of Rab1 (Tan et al., 2011; Goody et al., 2012). Thus, Rab1 is directly targeted and its activity is controlled by four different Dot/Icm effectors that catalyze different PTMs.

It is noteworthy that ubiquitination, mediated by the Dot/Icm effectors SidC and SdcA, could also participate to ER recruitment on the LCV. The Dot/Icm effector SidC and its paralog SdcA were proposed to function as vesicle fusion tethering factors involved in the recruitment of ER vesicles on the LCV (Luo and Isberg, 2004; Ragaz et al., 2008). Recently, infection by a WT *L. pneumophila* strain was shown to mediate the mono-ubiquitination of Rab1 on lysine 187 (Horenkamp et al., 2014). Given that cells infected with the double mutant *sdcA-sidC* did not exhibit this Rab1 PTM, it was assumed that Rab1 ubiquitination required the Dot/Icm effectors SidC and SdcA. However, ectopic expression of SidC or SdcA alone in HEK293 cells did not result in Rab1 ubiquitination, which suggests that neither SidC nor SdcA are E3 ubiquitin ligases. By contrast, another study demonstrated that the N-terminal domain of SidC exhibits ubiquitin ligase activity that catalyzes polyubiquitin chains formation and is necessary for ER recruitment on the LCV (Hsu et al., 2014). According to the authors, SidC does not seem to directly target Rab1 but more likely triggers a remodeling of proteins composition at the surface of the LCV. Although the mono-ubiquination of Rab1 would be mediated by an indirect unknown mechanism and that the substrates and impact of the SidC/SdcA-catalyzed polyubiquitination remains unclear, both these studies highlight the role of ubiquitination in ER recruitment on the LCV.

### UBIQUITINATION OF BAT3 COULD MITIGATE THE EFFECTS OF DISRUPTING NORMAL VESICULAR TRAFFICKING

*Legionella pneumophila* co-opts host vesicular trafficking during infection, in particular to recruit ER on the LCV surface. It can be assumed that some Dot/Icm substrates are translocated to protect host cells against the cytotoxic stress generated by the ER traffic hijacking. Among them, the Dot/Icm effector LegU1 contains an F-box domain and interferes with ubiquitin signaling. It can be integrated into the functional SCF1 complex that confers E3 ubiquitin ligase activity. It specifically targets the host chaperone protein BAT3, a key regulator of the ER stress response. LegU1 associates with BAT3 and mediates its polyubiquitination *in vitro* (Ensminger and Isberg, 2010). Moreover, another translocated *L. pneumophila* protein, Lpg2160, plays a role in this complex by binding both the SCF complex and BAT3. These results suggest that this multicomplex formation leads to BAT3 ubiquitination, probably to modulate the ER stress response during infection (Ensminger and Isberg, 2010).

## PTMs FOR SUSTAINING *Legionella* INTRACELLULAR REPLICATION

In the rough ER-like compartment of the LCV, *L. pneumophila* proliferates in a so-called replicative form until vacuolar nutrients become limiting. Polyubiquination of host cell proteins mediated by a *Legionella* effector has been recently proposed to be a bacterial strategy dedicated to generate sources of carbon and energy needed for microbial proliferation *in vivo* (Price et al., 2011). Indeed, in addition to the CAAX farnesylation motif described above, AnkB from *L. pneumophila* Philadelphia strain harbors two ankyrin (ANK) protein–protein interaction domains and a F-box domain. In both macrophages and protozoa, AnkB functions as a bona fide F-box protein where it recruits Skp1, thus subverting the host SCF1 complex and functionning as a platform for the docking of polyubiquitinated proteins to the LCV membrane. The polyubiquitinated proteins assembled by AnkB on the LCV are preferentially enriched for Lys48-linked polyubiquitinated proteins, which is a hallmark for proteasomal degradation, that generate 2–24 amino acid peptides (Price et al., 2011). Interestingly, substitution of Lys48 to Arg abolishes the decoration of the LCV with polyubiquitinated proteins and blocks intracellular proliferation. Moreover, inhibition of proteasome, or host amino- and oligo-peptidases that degrade the short peptides generated by proteasomal degradation, blocks intracellular proliferation (Price et al., 2011). However, both inhibitions are bypassed by excess amino acid supplementation. Together these data strongly support that AnkB promotes proteasome-mediated generation of free amino acids essential as energy and carbon sources for *L. pneumophila* intracellular proliferation (**Figure 2**).

It is noteworthy that in some *L. pneumophila* strains such as the strain Paris, AnkB does not contain the CAAX motif. Given they do not localize to the LCV, these AnkB homologues might not be key effectors of *L. pneumophila* that generate nutrients for intracellular growth. A yeast two-hybrid screen and co-immunoprecipitation analysis identified ParvB as one target of the *L. pneumophila* F-box protein AnkB encoded by strain Paris. ParvB, or affixin, is known to play important roles in focal adhesion, cell spreading and motility. Surprisingly, expression of AnkB led to a decrease of ubiquitination of ParvB. Thus, it was proposed that *L. pneumophila* modulates ubiquitination of ParvB by competing with eukaryotic E3 ligases for the specific protein–protein interaction site of ParvB. However, the role of AnkB in the infectious cycle of *L. pneumophila* strain Paris remains unknown (Lomma et al., 2010).

## PTMs FOR CONTROLLING HOST CELL GENES EXPRESSION

### PHOSPHORYLATION OF IκB FOR CONTROLLING THE NF-κB DEPENDENT GENES TRANSCRIPTION

After phagocytosis, *L. pneumophila* resides and replicates in the LCV within the host cytosol. Consequently, survival of the host cell is necessary for successful replication. To prevent cell death, some Dot/Icm translocated substrates interfere with pro-death pathways (Laguna et al., 2006; Banga et al., 2007). A second mechanism of preventing host cell death during infection is to stimulate the NF-κB pathway, which results in up-regulation of genes encoding anti-apoptotic proteins (Karin and Lin, 2002). NF-κB homo- and heterodimers are master transcription regulators of the mammalian innate immune response that control the expression of almost 400 genes (Karin and Lin, 2002; Ahn and Aggarwal, 2005; Hayden and Ghosh, 2008). NF-κB activation can result from sensing of pathogen associated molecular patterns (PAMPs) by the pattern recognition receptors (PRRs), which leads to activation of IκB kinases (IKKs). Once activated, IKKs phosphorylate IκB family members, inhibitory proteins that are bound to NF-κB subunits in the cell cytoplasm, thus triggering IκB ubiquitination, IκB degradation, and subsequent translocation of NF-κB into the nucleus (Hayden and Ghosh, 2008). *L. pneumophila* infection results in increased Dot/Icm-dependent transcription of NF-κB subunits as well as NF-κB regulated genes including pro-inflammatory cytokines and antagonists of apoptosis (Losick and Isberg, 2006; Abu-Zant et al., 2007; Shin et al., 2008). Besides the engagement of PRRs with PAMPs, direct targeting of the pathway by a Dot/Icm effector, namely LegK1, has been demonstrated (Ge et al., 2009; **Figure 2**). LegK1 efficiently phosphorylates IκB on Ser-32 and Ser-36 both *in vitro* and in cells, thus mimicking the host IKKs. Ectopic expression of the protein in mammalian cells results in activation of an NF-κB-dependent promoter. The kinase activity is necessary for this activation, as a point mutation in the ATP binding domain or a catalytic residue abolishes NF-κB activity (Ge et al., 2009; Losick et al., 2010), and cell-free reconstitution revealed that LegK1 stimulated NF-κB activation in the absence of IKKs (Ge et al., 2009).

### METHYLATION OF HISTONES FOR CONTROLLING HOST CELL GENE TRANSCRIPTION

*Legionella*-containing vacuoles are studded with an increasing number of ribosomes during the first 8 h after bacterial internalization, after which the bacteria start to replicate in the vacuole. Besides, transcription of rRNA genes (rDNAs) in the nucleolus is known to be regulated by epigenetic chromatin modifications including histone H3 lysine (de)methylation. Recently, the Dot/Icm LegAS4 from *L. pneumophila* Philadelphia strain was shown to localize in the host nucleolus and promoted rDNA transcription (Li et al., 2013; **Figure 2**). LegAS4 contains an active SET-domain-sharing 35% sequence identity with eukaryotic NSD2/3 Lys Histone Methyltransferases of the SET2 family. *In vitro* studies on histone H3 substrate, using methylation-specific H3 antibodies, show that LegAS4 catalyses dimethylation of histone H3 on Lys4 (H3K4me2). Consistently, ectopic expression of LegAS4 in human cells is associated with increased levels of H3K4me2 at rDNA promoters and the activation of the transcription of these genes. LegAS4’s association with rDNA chromatin is mediated by interaction with host HP1a/c. Docking of LegAS4 to these regions through binding to HP1, and subsequent methylation of H3K4, might convert the epigenetically silent state of rDNA genes to an active state methylated H3. Stimulation of rDNA transcription might contribute to bacterial replication in two flavors. The enforced higher proliferation potential of infected cells, resulting from activation of rDNA transcription, could provide a better niche for bacterial replication. On the other hand, intracellular bacteria could exploit host ribosome activity for its own survival advantages (Li et al., 2013).

Interestingly, mass spectrometry analysis revealed that the equivalent effector of LegAS4 from the *L. pneumophila* strain Paris, named RomA (for regulator of methylation A) trimethylates *in vitro* Lys14 of H3 (H3K14me3), a histone mark not previously described in mammals (Rolando et al., 2013b). This epigenetic mark was confirmed by systematic site-directed mutagenesis of the lysine residues in the N-terminal tail of H3. It is noteworthy that while H3 methylation was almost completely decreased when H3 was mutated on K14, RomA enzymatic activity appeared to be also reduced on H3 carrying a mutated K4. However, no H3K4 methylation was revealed in western-blot probed with anti-H3K4me2 or H3K4me3 antibodies, thus suggesting that RomA only targets K14 of H3 and that H3K4 methylation could influence H3K14 methylation by being part of the motif required by RomA to bind to its substrate (Rolando et al., 2013b). By promoting a burst of H3K14me3, RomA decreases H3K14 acetylation, which is an activating mark, thus leading to repression of host gene expression. In addition, ChIP-seq analysis identified 4,870 H3K14 methylated promoter regions, including at innate immune genes, during *Legionella* infection.

Recently, the H3K14-specific methylation was shown to be conserved in cells infected by seven different strains of *L. pneumophila*, including the Philadelphia 1 (Lp02) strain (Rolando and Buchrieser, 2014). Thus, there is more likely no different specificity of the methylation activities of LegAS4 and RomA, and despite slight discrepancies about the biochemistry and the biological effect of these effectors, both these studies highlight the key role of histone PTMs during *Legionella* infection (**Figure 2**).

### GLYCOSYLATION OF EF1A FOR INHIBITING THE HOST CELL TRANSLATION

In addition to controlling the host cell gene transcription, *L. pneumophila* is able to inhibit the overall host cell translation. *L. pneumophila* encodes three Dot/Icm effectors, namely Lgt1, Lgt2, and Lgt3, that monoglycosylate the serine residue Ser53 of the GTPase domain of the host translational factor eEF1A (eukaryotic Elongation Factor 1A), leading to the inhibition of protein synthesis, and consequently to the death of the host cell (Belyi et al., 2006). Although EF1A glycosylation seems to promote *L. pneumophila* pathogenesis, the biological role of this PTM remains to be addressed. Because their activities cause the host cell death, glysosyltransferases are usually considered like bacterial toxins rather than molecular tools that hijack host cell pathways to the benefit of the bacteria. However, it can be assumed that the inhibition of host cell protein synthesis leads to the overall decrease of the host metabolism, which promotes the ability of the bacteria to overcome the cellular response and consequently to replicate (Belyi et al., 2011). Moreover, it has been recently shown that Lgt1, Lgt2, Lgt3 plus two others Dot/Icm effectors, SidI and SidL that respectively, interacts with eEF1A and eEF1B (Shen et al., 2009) and inhibit protein synthesis by an unknown mechanism, are critical to control the host cell transcription response to *Legionella* infection (Fontana et al., 2011). In fact, these Dot/Icm effectors decrease the overall translation of host cell proteins, among which the NF-κB inhibitor IκB, thus promoting the activation of the NF-κB pathway. In that way, glycosylation of eEF1A by these effectors and thus inhibition of host cell translation could potentiate the activation of the NF-κB pathway, already controlled by the IκB phosphorylation by LegK1, as described above.

## CONCLUSION

Given PTMs play key roles in the cellular biology, it is not surprising that interference with host PTMs is a strategy widely used by bacterial pathogens to not only escape from host cell defences but also to hijack host cell pathways to their benefit. However, recent technological progresses in the detection of PTMs and advanced functional studies of the host–bacteria relationship highlighted an unexpected diversity of the PTMs triggered by bacteria and the complexity of these processes in host–pathogen interactions, thus making studies of bacteria-mediated PTMs an emerging field of research.

*Legionella pneumophila* is a paradigm of a pathogenic bacteria that evolved sophisticated biochemical strategies to successfully infect and replicate into professional bacteria killer phagocytic cells. In fact, *L. pneumophila* is a unique example for the co-evolution of a bacterium with environmental hosts, namely amoeba, that results in the acquisition of many genes encoding proteins that can be secreted by the Dot/Icm T4SS and trigger diverse PTMs into the host cells. Indeed, the large repertoire of Dot/Icm effectors enables the bacteria to phosphorylate, alkylate, ubiquitinate, glycosylate, AMPylate, and phosphocholinate specific host cell proteins. Noteworthy, *L. pneumophila* also catalyze PTMs of its own proteins, namely some of Dot/Icm effectors, to control their localization and/or their stability in the host cell, and subsequently their activity during the infection. Importantly, despite PTMs are usually catalyzed by eukaryotic-like proteins, some of them are performed by enzymes that do not exhibit similarity with their eukaryotic counterparts. More interestingly, research on Dot/Icm effectors functional roles lead to the discovery of a new PTM, namely the reversible phosphocholination, that may also be used by eukaryotic cells to modulate cellular functions, as previously suggested by studies that detected phosphoryl-choline substituted peptides secreted by nematodes and from mammalian cells residing in the placenta (Lovell et al., 2007; Grabitzki et al., 2008). AMPylation had been also discovered by studying infections by *V. parahaemolyticus* and *H. somni*, a human pathogen and the causal agent of septicemia in cattle, respectively (Worby et al., 2009; Yarbrough et al., 2009). These discoveries reveal that studies of the relationship between pathogenic bacteria and their host cells could lead the way to improve our knowledge of the eukaryotic PTMs and complex cellular processes that are associated to.

Interestingly, *L. pneumophila* targets host proteins that have been already described to be preferential targeted for bacterial-induced PTMs. This is the case of regulators of the NF-κB pathway, which allows the bacteria to control both anti-apoptotic genes and host immune response, like previously demonstrated for *Shigella flexneri* (Kim et al., 2005), *Salmonella typhimurium* (Le Negrate et al., 2008), *L. monocytogenes* (Gouin et al., 2010), and *Yersinia* species (Mittal et al., 2006). Moreover, *L. pneumophila* joins those bacteria that secrete effectors manipulating PTMs at histones tails, allowing a fine-tuned regulation of host genes transcription (Hamon and Cossart, 2008; Bierne, 2013; Rolando et al., 2013a). These recent insights highlight the key role of both these processes and their control by PTMs in the pathogenic bacteria–host relationships.

## Conflict of Interest Statement

The authors declare that the research was conducted in the absence of any commercial or financial relationships that could be construed as a potential conflict of interest.
